# Smokeless tobacco use and public health nutrition: a global systematic review

**DOI:** 10.1017/S1368980022001331

**Published:** 2022-05-27

**Authors:** Shikha Saxena, Prashant Kumar Singh, Lucky Singh, Shekhar Kashyap, Shalini Singh

**Affiliations:** 1 Division of Preventive Oncology, National Institute of Cancer Prevention and Research, Indian Council of Medical Research, Noida, Uttar Pradesh 201301, India; 2 ICMR National Institute of Medical Statistics, New Delhi, India; 3 Department of Cardiology, Army Research & Referral Hospital, New Delhi, India

**Keywords:** Smokeless tobacco, BMI, Nutrition, Food insecurity, Metabolic disorders

## Abstract

**Objective:**

Tobacco consumption among low- and middle-income countries where food insecurity remains a challenge poses several concerns. This review examines the available global evidence linking smokeless tobacco (SLT) use with public health nutrition and its implications.

**Design:**

Systematic review of articles extracted from PubMed and Scopus from January 2000 to December 2020.

**Setting:**

Included studies that demonstrated the relationship between SLT and nutrition-related factors, that is, BMI, malnutrition, anaemia, poor birth outcomes and metabolic disorders. Preferred Reporting Items for Systematic Reviews and Meta-Analysis (PRISMA) guidelines have been followed to conduct the systematic evidence review.

**Participants:**

A total of thirty-four studies were finally used in the systematic review, which included cross-sectional (thirty-one) and cohort (three).

**Results:**

SLT use has a huge impact on body weight, alteration in taste, poor oral health, and consumption of fruits and vegetables leading to malnutrition. Maternal use of SLT not only leads to anaemia but also hampers birth outcomes. Increased risk of metabolic syndrome and gallstone disease among SLT users are also well documented in the studies.

**Conclusion:**

The review highlights the linkages between SLT usage and poor nutritional outcomes. Tobacco control efforts should be convergent with public health nutrition to achieve overall health benefits. Attention is also required to explore suitable mechanisms for SLT cessation combined with enhancing food and nutrition security at the community level in sync with investments in public health nutrition intervention.

The consumption of smokeless tobacco (SLT) has been reported in 127 countries^(1)^. As per the Comprehensive Smokeless Tobacco Health Education Act (CSTHEA), under the Centre for Disease Control and Prevention (CDC), SLT is defined as ‘any finely cut, ground, powdered, or leaf tobacco that is intended to be placed in the oral cavity’ (CDC). SLT comes as either snuff or chewing tobacco. More than 85 % of the SLT-related burden is present in South and South-East Asia, followed by Africa^(1)^. When compared to smoking, SLT use was found to be higher among women in many low- and middle-income countries mainly due to its social acceptability and the notion of it being less harmful than smoking^(2)^. About 2·5 million disability-adjusted life years and 90 791 lives were lost globally due to oral, pharyngeal and oesophageal cancers owing to SLT use^(1)^. Though studies have also shown adverse health impacts of SLT including cardiovascular and reproductive morbidities amongst both men and women^(3)^, so far no attempts have been made to understand the linkages with food insecurity and nutrition. This study examined the available global shreds of evidence that links SLT use with public health nutrition and food insecurity and its possible implications on malnutrition, poor birth outcomes and metabolic disorders.

There have been a plethora of evidence-based researches that study the impact of smoking on diet, dietary antioxidants, body weight and metabolic disorders^(4–10)^. Some previous studies have also shown linkages between smoking and malnutrition in developing countries. For instance, a survey amongst the low- and middle-income households in rural Indonesia reported that households comprising one smoker spent less on food budget (68 %) as compared to the households with non-smokers (75 %)^(11)^. Habitual smoking by parents not only hampers the quality but also the quantity of the food consumed by the poorest households, which further leads to significant fall in the nutritional status of children in these households^(11)^.

The available literature is deficient in studies that links SLT and nutrition despite its well-established linkages with various cancers^(12)^ and non-communicable diseases^(13)^. Particularly lacking is an understanding of SLT control as an important development issue and its linkages to the food and nutrition discourse. There was thus a clear and significant information gap in the existing literature on SLT use and its implication on nutritional outcomes based on the global evidence.

## Methods

### Protocol and guidelines

We followed the guidelines of Preferred Reporting Items for Systematic Reviews and Meta-Analysis (PRISMA) checklist for the elaboration of this systematic review^(14)^. The systematic review entails evidence on articulated questions that use systematic and explicit methods to identify, select and critically assess relevant primary research, and to extract and analyse data from the studies included in the review (*Systematic Reviews: CRD’s guidance for undertaking reviews in health care*, 2009)^(15)^. This review examines the highest level of evidence by combining the relevant data from the existing literature to derive informed decisions. The review has constructed themes based on the reported evidence and synthesised qualitative summary tables. The systematic review protocol has been registered with PROSPERO International’s prospective register of systematic reviews (registration number: CRD42021253694). The review was formulated to address mainly two research questions: (1) What is the impact of SLT on nutritional outcomes which includes body weight, malnutrition, anaemia and birth outcomes? and (2) What is the impact of SLT on metabolic disorders?

The preliminary conceptual framework that is used has given information on the development and segregation of many domains that had been impacted by SLT. This was seen after triangulating the findings of the thirty-four review papers. In generating the framework, a panel of experts panel considered themes and concepts identified under each of the categories from the evidence systematic review, post which, the authors structured and reorganised concepts under main health, functioning and conceptually related domains. This framework provided the basis and insights to comprehend the impact of SLT on health and functioning and conceptually related domains^(16)^.

### Database and search strategy

The total number (*n* 1880) of research papers were extracted from an electronic advance search of PubMed and Scopus published from January 2000 to December 2020 for review. Search criteria included MeSH terms with ‘smokeless tobacco’ and ‘underweight’, ‘anemia’, ‘BMI’, ‘diet’, ‘food’, ‘food security’, ‘fruit’, ‘hunger’, ‘malnutrition’, ‘obesity’, ‘vegetables’, ‘hunger’, ‘nutrition’, ‘fruits’ and ‘vegetables’.

### Inclusion and exclusion criteria

**Fig. 1:**
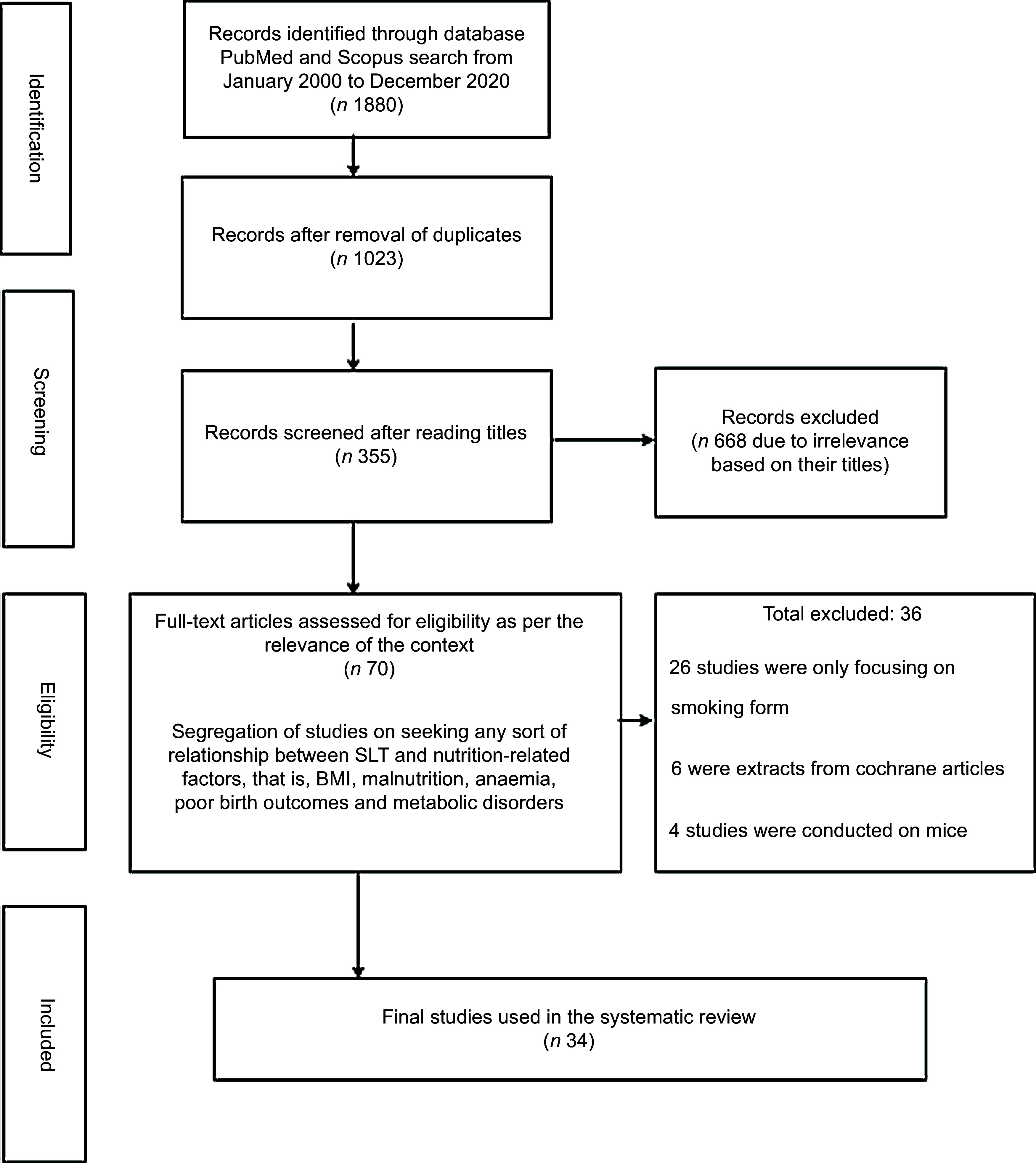
The PRISMA flowchart for selection of epidemiological studies. PRISMA, Preferred Reporting Items for Systematic Reviews and Meta-Analysis; SLT, smokeless tobacco

Research articles were then selected after discerning relevant literature pertaining to the underlying objective. Organisation of research articles was done on seeking any sort of relationship between SLT and nutritional outcomes like BMI, malnutrition, anaemia, poor birth outcomes and metabolic disorders (*n* 355). The search strategy is shown in Fig. 1. A total number of thirty-four papers were finally used in the systematic review (Tables 1 and 2).

**Table 1:** Summary of the selected studies used in systematic review (high-income countries)

S.no	First author	Year	Study design	Age groups	Aim/objectives	Place of origin
1	Hansson *et al.*	2011	Cross-sectional	18–84 years	To investigate the relationship between the use of snus, weight gain (≥5 %) and the incidence of obesity (BMI ≥ 30 kg/m^2^).	Stockholm, Sweden
2	Wilson *et al*.	2016	Cross-sectional	Mean age of 70·2 years	To study snus use, smoking and survival among prostate cancer patients.	Sweden
3	Liu *et al.*	2016	Cross-sectional	–	To study the effect of SLT on oral bacteria.	Little Rock, Arkansas
4	Lund *et al.*	2018	Cross-sectional	18–45 years	To investigate the associations between BMI and female snus use, and between self-rated general health and female snus use.	Norway
5	Vander Wag *et al.*	2005	Cross-sectional	20 years	To study the association between smokeless tobacco (SLT) use and body weight among 22 974 Air Force recruits.	Rochester, New York
6	Accortt *et al.*	2005	Cross-sectional	25–74 years	To study SLT as a risk factor for oral leukoplakia and oral cancer, and the cancer risk factors associated with its use.	Birmingham, Alabama
7	Rodu *et al.*	2004	Cross-sectional and prospective follow-up study	25–64 years	To explore the effect of tobacco use (smoking tobacco and SLT) and cessation on body weight.	Northern Sweden
8	Carlsson *et al.*	2017	Cohort	Mean age of 47 years	To explore the association between snus use and risk of type 2 diabetes using pooled data from five cohorts.	Vasterbotten, Northern Sweden
9	Quandt *et al.*	2005	Cross-sectional	60 years and older	To identify risk factors for low bone mineral density among older women in a multi-ethnic population, with particular attention to smoking and SLT use.	Robeson County, North Carolina
10	Sundbeck *et al.*	2009	Cross-sectional	30–75 years	To describe the consumption of snuff in a rural male population and to explore associations between snuff use and obesity.	Vara Municipality, Western Sweden
11	Ryman *et al.*	2018	Cross-sectional	–	To characterise the associations between iq’mik use and biomarkers of cardiometabolic status.	Alaska, USA
12	Westra *et al.*	2018	Cross-sectional	Mean age of 66 + 12 years	To investigate the effect of SLT, cigar and/or pipe smoking on the development of Barrett’s oesophagus in White male patients with gastroesophageal reflux disease.	Amsterdam

**Table 2:** Summary of the selected studies used in systematic review (low-income and middle-income countries)

S.no	First author	Year	Study design	Age groups	Aim/objectives	Place of origin
1	Razzaque *et al.*	2011	Cross-sectional	25–64 years	To examine non-communicable diseases risk factors among adults 25 to 64 years old of the Matlab Health and Demographic Surveillance System using WHO STEPwise methodology.	Bangladesh
2	Blankson *et al.*	2012	Cross-sectional	>60 years	To describe the anthropometric and physical status of a sample of elderly women in rural Ghana and examine factors associated with a low BMI.	Ghana
3	Block and Webb	2009	Cross-sectional	–	To study the impact of expenditure on smoking products in low-income households on child nutrition, as mediated via reduced food expenditure.	Indonesia
4	Dhamnetiya *et al.*	2019	Cross-sectional	20 years and above	To find correlates (demographic, dietary and behavioural) of gall stone disease in patients attending teaching hospitals in North India.	New Delhi, North India
5	Diendere *et al.*	2020	Cross-sectional	≥18 years	To assess the prevalence of SLT use and its associated factors among rural women in Burkina Faso by using nationally representative data.	Burkina Faso, West Africa
6	Sinha *et al.*	2004	Cross-sectional	School students aged 13–15 years	To study attitude and behaviour towards the use of tobacco products in twenty-five randomly selected villages.	Mizoram and Churachandpur, India
7	Ganganahalli *et al.*	2017	Cross-sectional	20–25 years	To estimate cotinine levels among pregnant women using and not using SLT (Mishri) and to correlate cotinine level with anthropometry of newborns.	Maharashtra, India
8	González-Rivas *et al.*	2017	Cross-sectional	20 years and older	To evaluate the relationship between chimó use and type 2 diabetes in a population with high prevalence of SLT use in the Andes region of Venezuela.	Venezuela, South America
9	Shafique *et al.*	2013	Cross-sectional	16–75 years	To determine the relationship of raw areca nut and areca nut chewing with tobacco additives and metabolic syndrome.	Karachi, Pakistan
10	Mustufa *et al.*	2017	Cross-sectional	Primary school children aged 7–11 years	To determine the health status of primary school children in seven tehsils of district Lasbela, Balochistan.	Lasbela, Balochistan
11	Senn *et al.*	2009	Cross-sectional	Mean age of 26 years	To investigate the habits of betel nut chewing and possible impact on pregnancy.	Papua New Guinea
12	Nonterah *et al.*	2018	Cross-sectional	40–60 years	The study characterised the sociodemographic and behavioural factors influencing BMI among adults.	Northern Ghana
13	Sinha *et al.*	2018	Cross-sectional	15 years and above	To study the global burden of SLT use among adults was estimated using nationally representative data of countries by gender and country income group.	Data from 140 countries
14	Pednekar *et al.*	2008	Cohort	>35 years	To study the joint effects of tobacco use and body mass on all-cause mortality in Mumbai.	Mumbai, India
15	Pednekar *et al.*	2006	Cross-sectional	>35 years	To study association between tobacco use and BMI in urban Indian population.	Mumbai, India
16	P. Geldsetzer et al	2019	Cross-sectional	Men: 15–54 years; women: 15–49 years	To study the national prevalence of anaemia among men in India.	India
17	Gupta Sreevidya	2004	Prospective cohort study	>35 years	To study the effect of using SLT during pregnancy on babies’ birth weight and gestational age at birth.	India
18	Pratinidhi *et al.*	2014	Cross-sectional	15–49 years	To compare the outcome of pregnancy among women using Mishri during pregnancy and those not using it at Krishna hospital, Karad.	Maharashtra, India
19	Mohandas *et al.*	2019	Cross-sectional	15–49 years	To study nutritional status and its associated factors among tribal women in the reproductive age group.	Wayanad, Kerala
20	Sreeramareddy and Ramakrishnareddy	2017	Cross-sectional	15–49 years	To find out the association between tobacco and food insecurity.	Nepal
21	Subramoney and Gupta	2008	Cross-sectional	20 years and above	To study the effect of SLT use on the haemoglobin levels in a population-based cohort of pregnant women.	India
22	Virk-Baker *et al.*	2019	Cross-sectional	–	To analyse and compare the consumption of major food categories, that is, cereal grains, legumes and beans, fish, meat, milk, fruit, vegetable, sugar, eggs, oil/fat, and beverages among tobacco use (including smoking tobacco and SLT) and non-use households.	Bangladesh

SLT, smokeless tobacco.

Following was the inclusion criteria: (i) primary research papers published from 2000 to 2020 in English language and (ii) exposure variable: SLT. Studies that encompassed smoking were considered only if SLT had contributed to the outcome in combination with smoking; (iii) outcome variable: nutrition-related factors, that is, BMI, body weight, malnutrition, anaemia, poor birth outcomes and metabolic disorders; and (iv) study design: cross-sectional, cohort and review papers.

Following was the exclusion criteria: (i) studies that solely focused on smoking conducted on mice were excluded; and (ii) extracts from Cochrane articles, case reports and letters were also excluded.

## Results

### Characteristics of the included studies

The selected studies comprised cross-sectional (thirty-one) and cohort (three) studies. Half of the studies were from the South-East Asia region (seventeen), out of which eleven were from India followed by Bangladesh (two), Pakistan (two) and Nepal (one). Six studies were included from Scandinavian countries which were based on the impact of snus use. Five studies were from the USA and three were from African countries. These studies represented age diversity with the inclusion of primary school children, teenagers and adults (13–60 years of age). Studies described the general population with different demographic and socio-economic conditions.

### Smokeless tobacco, food expenditure and food insecurity

The cause of food insecurity is not merely access and consumption but adequate absorption of the nutrients in adequate quantity and quality^(17)^. For studying the association between tobacco and household food insecurity, a study from Nepal reported a higher household food insecurity among households where men either smoked or consumed SLT as compared to those households where none of the members either smoked or consumed SLT^(18)^. In the USA, a study based on National Health and Nutrition Examination Survey (NHANES) from 1999 to 2014 showed that not only smoking but also the usage of alternative tobacco products including SLT has been significantly associated with household food insecurity^(19)^.

A household survey on tobacco and food expenditure patterns in Bangladesh reported that the tobacco expenditure was quite high in households with dual-tobacco users (smoking and smokeless) as compared to expenses incurred on cooking oil and other fat items^(20)^. Further comparisons were made on the basis of food energy gain on a daily basis if the money was spent to purchase cereals among the smoking-only, smokeless-only and dual-tobacco user households. A potential gain of daily food energy found was 857, 437 and 1512 kcals, respectively^(20)^. Another study from Mumbai, India, found higher daily expenditure on *gutka* (a form of SLT) as compared to food items among street children aged 6 to 8 years^(21)^. While choosing tobacco over food, the study noted that these children were not only limiting their options to a healthy diet but also suffering from its ill effects, that is, cough, sore throat, weakness and breathing problems^(21)^.

### Smokeless tobacco and BMI

There is adequate evidence of nicotine cessation leading to weight gain among smokers due to the regulation of appetite^(22)^, but there is hardly any literature on the proven linkage between the interference of SLT use and appetite or dietary outcomes. However, the reviewed literature demonstrated evidence of change in the weight and thus BMI with SLT use. For instance, a study from the USA observed that users consuming SLT for more than 30 d were found obese. Likewise, the chances of being categorised as overweight was higher for daily (OR = 1·29, 95 % CI (1·07, 1·54)), occasional (OR = 1·50, 95 % CI (1·17, 1·93)), former (OR = 1·33, 95 % CI (1·05, 1·67)) and experimental (OR = 1·13, 95 % CI (1·02, 1·24)) SLT users relative to non-SLT users^(23)^. There have also been evidence of increased weight (≥5 %) (OR = 1·31, 95 % CI (1·04, 1·65)) and incidence of obesity (BMI ≥ 30 kg/m^2^) (OR = 1·93, 95 % CI (1·13, 3·30)) after adjustment of other factors in males with snus use in the USA^(24)^. Weight gain has been reported among the former smokers who substituted smoking with snuff^(25)^.

Among women, SLT use was linked with lower BMI in northern rural Ghana^(26,27)^. In a cross-sectional population-based sample among female snus users in Sweden, daily snus use was found to be associated with lower chances of being overweight and higher chances of being underweight and worse general health^(28)^. Tobacco consumed in different forms also has an impact on weight. A population-based cohort study in Mumbai, India, found that men and women using different types of SLT had BMI less than 16·0 and had approximately twice the risk of death as compared to the non-users^(29)^.

### Smokeless tobacco and malnutrition

Based on BMI scores, a higher prevalence of malnutrition co-existing with poor oral health was reported amongst the primary school children consuming SLT in Pakistan^(30)^. Similarly, a higher prevalence of undernutrition was found among those tribal women who were SLT users in Wayanad, India^(31)^. Low consumption of fruits and vegetables (90 % below recommended levels) has been found among the SLT users in Bangladesh^(32)^. While examining the relationship between smoking and diet, it has also been also reported that tobacco users should be counselled to increase their intake of fruits, vegetables and high-fibre grains as they substantially have low intakes of foods related to cancer risk^(33)^. Increased undernourishment, dental problems and high blood pressure were also seen among the rural women consuming SLT in Burkrina Faso^(34)^.

SLT is also being consumed due to a common misbelief of promoting oral health and benefits to teeth. Tobacco containing dentifrices in the form of toothpaste, tooth powder roasted and powdered tobacco, etc., are widely available in the markets. Some people in the north-eastern parts of India also believe that tobacco water (tuibur) protects from the bites of insects and possesses antiseptic and anti-snake venom properties^(35)^. The use of such aqueous tobacco extracts has shown to affect the growth of some oral bacterial species that influence the healthy ecological balance of oral bacteria in humans. The growth and viability of these species were found to be affected in a concentration-dependent manner. The growth of these bacterial strains thus varies due to incongruence between nutrients and toxic compounds in SLT products^(36)^. It is well understood that anthropometric data, BMI and skinfold thickness are significantly related to bone mineral density. The bone mineral density is a reflection of the nutritional status of women, and osteoporosis is higher among the malnourished^(37)^. While the decrease in bone mineral density is evident with ageing, the decline was greater for women who were SLT users as compared to those who never had SLT in North Carolina^(38)^.

Alteration in taste has also been reported with the use of SLT which could also be one of the reasons for malnutrition. Another study has reported a significantly higher intake of cereals (*β* = 152·46, *P* < 0·0001 sugar (*β* = 8·16, *P* < 0·0001) and lower consumption of dairy and milk products (*β* = -17·11, *P* < 0·01) and oil/fat (*β* = -10·30, *P* < 0·01) in households consuming any form of tobacco (smoke, smokeless or dual form)^(20)^. SLT has also been associated with low BMI and low haemoglobin levels in Fe deficiency anaemia among women^(39,40)^. Fe deficiency anaemia is not just limited to women SLT users but also men SLT users^(41)^.

### Smokeless tobacco and poor birth outcomes

Initiation of SLT usage can occur during pregnancy to relieve symptoms associated with high physiological stress, that is, nausea, vomiting and constipation. The pooled prevalence of SLT use among pregnant women was highest in the South-East Asia region (2·6 %) and lowest in Europe (0·1 %). SLT use during pregnancy also increases the likelihood of stillbirth in low-income countries^(42)^. SLT use during pregnancy is also associated with low haemoglobin levels (10·00 g/dl or less) among the population-based cohort of 918 women in Mumbai^(40)^. Mean haemoglobin (g %) was found significantly lesser (*t* = -15·24, *P* = 0·000) among pregnant users of *Mishri* (a form of SLT) (10·4 ± 0·90) compared to non-users (11·6 ± 1·05)^(43)^. In another prospective cohort of 1217 women in Mumbai, India, SLT use has been linked to a decrease in gestational age as well as the significant decrease in birth weight^(44)^.Similarly, 81 % of pregnant women users that were SLT users delivered babies with birth weight of less than 2·5 kg. Complications like oligohydramnios and fetal distress were found to be significantly more among the SLT users^(43)^. Moreover, the cotinine levels among users were found to be negatively correlated with anthropometric measurements of newborn babies^(45)^. In a study conducted in Papua New Guinea, initiating betel nut chewing and low BMI during pregnancy have resulted in significant birth weight reduction^(46)^. The primary reasons listed for chewing betel nuts were to prevent morning sickness, halitosis and addiction^(46)^.

### Smokeless tobacco and metabolic disorders

A higher odds of developing Barrett’s oesophagus was found when a cigar or pipe was used in combination with SLT^(47)^. Snus used in the Scandinavian countries has been linked with the high risk of type 2 diabetes^(48)^. Similarly, Chimo (SLT) increased the odds of type 2 diabetes associated with low-fat mass in an Andean Venezuelan population by 77 %, representing a significant public health problem^(49)^. Despite the abundant consumption of marine-based food and physical activity which are considered to be cardioprotective, the use of Yup’ik (a form of SLT) may increase cardiometabolic risk amongst the Alaska Native people^(50)^. Evidence of snuff use and its association with CVD is conflicted. Swedish moist snus has not been proven to be associated with the heightened risk of fatal myocardial infarction^(3)^. However, a significant and positive association was found between metabolic syndrome and areca nut chewers with SLT additives^(51)^. A population-based longitudinal study in Sweden reported a positive independent association between metabolic syndrome and snus use. High doses of snus not only increases the risk of metabolic syndrome and hypertension but also has an effect on obesity and hypertriglyceridemia^(52)^. Besides physical inactivity, non-vegetarian diet, high intake of fat, family history and SLT consumption were also found to be some of the predisposing factors for the development of gallstone disease in North India^(53)^.

## Discussion

**Fig. 2:**
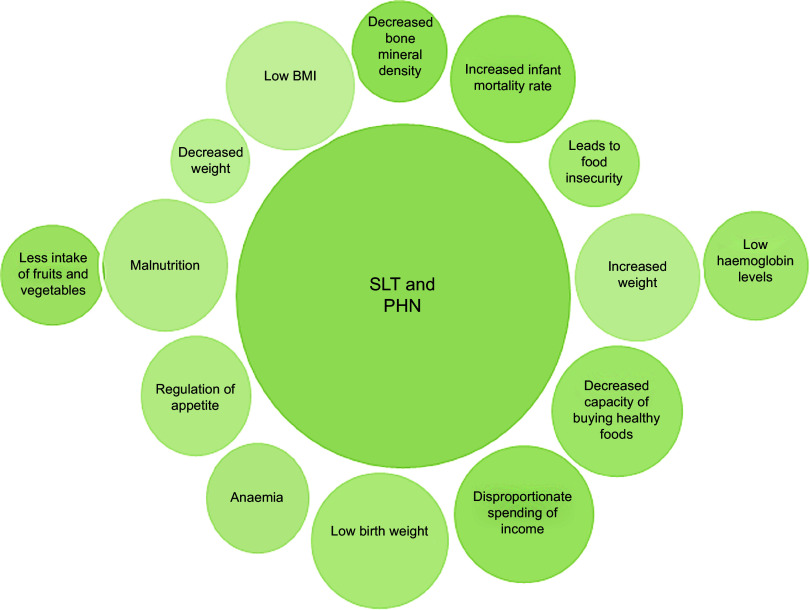
Diagrammatic representation of the association between smokeless tobacco use and public health nutrition; SLT, smokeless tobacco

To the best of our knowledge, this is the first systematic review to ascertain the linkage between SLT consumption and public health nutrition. Our findings suggest that SLT consumption is linked with various dimensions of public health nutrition (Fig. 2). Several public health and nutritional outcomes are mentioned in Table 3. These include nutritional, oral health, poor birth, behavioural and metabolic outcomes. SLT consumption is widespread in more than half of the countries globally. Synthesised results show that the use of SLT products leads to increased household food insecurity. Further, the household’s expenditure on tobacco outweighs the expenditure made on oil and fat. Increased intake of cereals and sugar and lower consumption of milk and fat products were seen in households consuming SLT or any other form of tobacco^(20)^. This may not only lead to decreased total energy intake but also compromise on the concept of a healthy eating plate or healthy diet. Reduction in perceived intensity of salt, sour and bitter taste leading to an aberration in the dietary pattern has been documented among SLT users^(54)^. In addition to this, greater alcohol intake and lower consumption of carbohydrates, confections and sweets, fruits and grains were also seen among the SLT users^(54)^. Therefore, alteration in taste, poor oral health and low consumption of fruits and vegetables associated with SLT use can be responsible for malnutrition. The differences in the food consumption pattern among the SLT and non-SLT users highlight the urgent need to address SLT usage as one of the underlying causes of food insecurity, malnutrition and poor nutritional outcomes. Hence, the inclusion of SLT cessation is crucial in the context of nutritional interventions.

**Table 3:** Public health and nutrition-related outcomes identified from the evidence systematic review

Nutritional outcomes	High food insecurity, increased and decreased weight leading to change in BMI, malnutrition, less intake of fruits and vegetables and decreased bone mineral density.
Oral health outcomes	Alteration in taste, regulation of appetite, impact on the healthy ecological balance of oral bacteria, gingival, periodontal inflammation and dental caries.
Poor birth outcomes	Increased infant mortality rates, low haemoglobin levels in anaemia and low birth weight.
Behavioural outcomes	Decreased capacity of buying healthy foods or disproportionate spending of income.
Metabolic outcomes	Barrett esophagus, risk of type 2 diabetes and gallstone disease.

Malnutrition and its relation to SLT use have been reported among South-East Asian countries. Women who used any form of SLT had lower BMI(<16) and had twice the risk of death when compared to non-SLT users^(29)^. Anaemia due to SLT use is not just limited to women, but it concurs in men as well^(41)^. Studies from both low middle income countries and high income countries support that different SLT products have an insignificant impact on fetal growth and increased risk of preterm delivery^(55)^. Low birth weight is another associated consequence of the consumption of SLT among pregnant women^(40,44,46)^. In order to reduce exposure to SLT before and during pregnancy, there is a need to develop culturally appropriate behavioural interventions in low-income countries^(56)^. Attention should be given to reduce SLT use as a component of routine antenatal care in order to minimise the adverse perinatal outcome^(43)^.

The review also confirms linkage of SLT use to loss of weight^(28)^ and also to weight gain^(24)^. Evidence of weight gain (≥5 %) and incidence of obesity (BMI ≥ 30 kg/m^2^) in males with snus use has been reported in the USA. However, BMI as a measure of body composition has limitations due to the exclusion of body fat or muscle mass^(24)^. One of the studies focused on the relationship between snus use and obesity depending on three different parameters (BMI ≥ 30, waist-hip-ratio ≥1·0 and waist circumference >102 centimetres). It was found that only waist-to-hip ratio is positively associated with snus use^(57)^. The possible explanation of the co-occurrence of obesity and high prevalence of SLT in rural areas was due to effective nicotine delivery system^(58)^, body image, physical activity and diet^(58)^. Moreover, no information was gathered on eating habits including the consumption of fruits and vegetables and frequency of eating^(59)^. Therefore, weight gain or loss remains an important question to investigate in future studies. Metabolic disorders, including type 2 diabetes, increased cardiometabolic risk, myocardial infarction, gallstone disease and Barrett’s oesophagus, are the other comorbidities that occur with the consumption of SLT^(3,47,48,50,53)^. Several studies in the past have also anticipated the metabolic effects of SLT use due to the sustained exposure to nicotine on the central nervous system as in the case of cigarette smoking^(60,61)^.

SLT usage also affects the diverse and composite human oral microbiome. The growth of some bacterial species is affected by SLT usage. The SLT aqueous extracts enhance the growth rate of some bacterial species which interferes with the opportunistic pathogens. These interactions potentially cause oral diseases like gingival and periodontal inflammation and dental caries. It is crucial to identify the toxicological effects of SLT products on the oral microbiota and the genotoxicity of their metabolites produced by oral microbiota^(36)^. Further studies are also warranted to identify the impact of various brands of SLT on oral bacterial species which could hamper the oral bacterial physiology and metabolism^(62)^. The decrease in the bone mineral density with ageing was more prominent among elderly women who consumed SLT, which distinctly shows the influence of SLT on their nutritional status. With the available evidences^(32,33)^ of low consumption of fruits and vegetables among SLT users, there is a need to do further research on the relationship between SLT and diet.

## Conclusion

To conclude, this study highlights the linkages between SLT usage and poor nutritional outcomes. These interplaying factors are crucial and need to be understood well for planning comprehensive interventions. Tobacco control research should be conducted in convergence with nutrition research for overall health benefits^(39)^. The review suggests the integration of public health nutrition programmes and tobacco cessation efforts. It also provides a clear picture of how SLT usage is linked to poor nutritional outcomes. Attention is needed to find appropriate mechanisms for the inclusion of SLT cessation in nutrition-sensitive programmes at the community level. Hence, both the issues should be seen together to arrive at a comprehensive solution in order to attain public health goals.

One of the limitations of this review is that PubMed and Scopus were the only search engines used which may exclude relevant papers not retrievable through it. We adhered to include articles from PubMed and Scopus, because these are proven sources of highly regarded scientific journals. The included studies were heterogeneous in nature due to the differences in the study outcomes, quality and designs. Results obtained should be carefully interpreted as the studies involved were not uniformly distributed across the countries.

## Supplementary material

For supplementary material accompanying this paper visit https://doi.org/10.1017/S1368980022001331. click here to view supplementary material
