# Supplementary material for: Smokeless tobacco use and public health nutrition: a global systematic review
Source: Public Health Nutr. 2022 May 27;26(1):46–55. doi: 10.1017/S1368980022001331 (PMC11077452; doi:10.1017/S1368980022001331)
Supplement: Supplementary file 1 [file S1368980022001331sup001.docx]

**Appendix 1**

**Table 4 Types of SLT products used across countries**

| Countries | Type of SLT used | As described in studies |
| --- | --- | --- |
| United States of America | Moist snuff is referred as Snus | a form of moist, smokeless tobacco popular in Sweden |
|  | Iq’mik | iq'mik, a traditional form of smokeless tobacco used by >50% of Yup'ik adults |
| Sweden | Snus | Not mentioned |
| Bangladesh | Tobacco leaf, goul, noshi, and zarda | Not mentioned |
| India | Creamy snuff (tooth paste) and tooth powder | Not mentioned |
|  | Gul | a pyrolysed tobacco product |
|  | Mishri | Roasted and powdered tobacco leaves applied to the gums using a finger. The principal constituent include alkaloid and nicotine being 1 to 7%. |
|  | Dry snuff (bajjar or tapkir) | Not mentioned |
|  | Gudakhu | Paste of tobacco and molasses |
|  | Tuibur or hidakphu | Manufactured by passing tobacco smoke through water, that’s is used for gargling not drinking |
|  | Betel quid | Combination of betel leaf, areca nut, slaked lime, tobacco and other condiments |
|  | Gutka | An industrially manufactured smokeless tobacco product containing tobacco, areca nut, slaked lime (calcium hydroxide), condiments, flavoring agents, and preservatives |
| Pakistan | Areca nut | Not mentioned |
| Nepal | Snuff and chewing tobacco | Not mentioned |
| Ghana | Snuff and chewing tobacoo | Not mentioned |
| Venezuela | Chimo | Not mentioned |
| Papua New Guinea | Betel quid | Not mentioned |
